# Necrotizing Infection of the Aortic Arch: Reconstruction Utilizing Unusual Extra-anatomic Bypass Grafts to Reroute Cerebral Blood Flow

**DOI:** 10.1055/s-0039-3401012

**Published:** 2020-02-04

**Authors:** Jonathan M. Hemli, Byron D. Patton, S. Jacob Scheinerman, Derek R. Brinster

**Affiliations:** 1 Department of Cardiovascular and Thoracic Surgery, Lenox Hill Hospital/Northwell Health, New York, New York

**Keywords:** extra-anatomic bypass, infective aortitis, homograft

## Abstract

Destructive infections of the aortic arch and great vessels are challenging to manage. We describe a novel technique for debranching the right cerebral and upper extremity arteries via composite extra-anatomic bypasses from the femoral artery, with subsequent homograft in-line reconstruction of the arch, in a patient with *Staphylococcus* sepsis and necrosis of the arch and great vessels.

## Introduction

Mycotic infection of the aorta can be managed utilizing open and/or endovascular techniques, 1 and not uncommonly involves a combination thereof. Although well described in the thoracic and abdominal aorta, and in the setting of contaminated graft material, primary bacterial infection isolated to the native aortic arch is reported less commonly. 2 Anatomic reconstruction of the arch in the setting of extensive necrosis that precludes an endovascular-focused approach can be technically challenging, not only because of the tissue destruction itself, but also secondary to the need for complex intraoperative circulatory management strategies to preserve cerebral, visceral, and spinal cord perfusion.

We report an unusual technique of maintaining cerebral blood flow utilizing sequential extra-anatomic bypasses based on the femoral artery, coupled with in-line homograft reconstruction of the arch, in a case which necessitated radical debridement and replacement of the entire aortic arch.

## Case Presentation

**Fig. 1:**
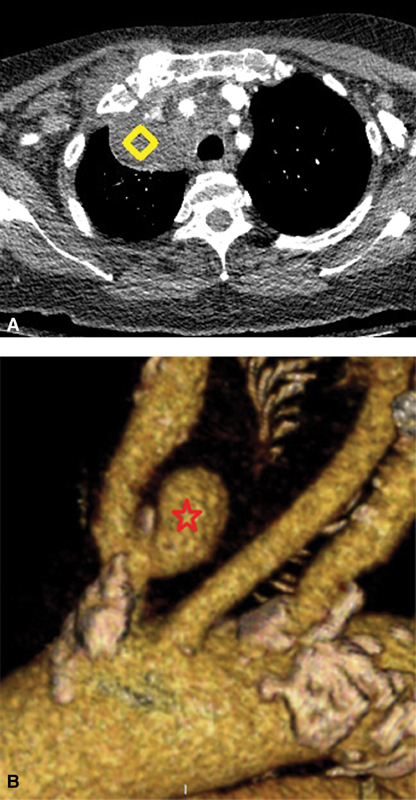
( **A, B** ) Phlegmon (diamond) surrounding the innominate artery, with mycotic pseudoaneurysm (star).

An 81-year-old female presented with malaise and dyspnea. A mass was evident within the superior mediastinum, surrounding the innominate artery, with extension into periaortic soft tissue ( Fig. 1A ). Blood cultures were positive for *Staphylococcus aureus.* The working diagnosis was mycotic pseudoaneurysm of the innominate artery ( Fig. 1B ), with extension into the periaortic space and threatened rupture. She was referred for surgical reconstruction.

The initial phase of the operation was directed at debranching the right cerebral and upper limb vessels. An 8-mm prosthetic Propaten graft (W L Gore & Associates, Inc., Flagstaff, AZ), originating from the right common femoral artery, was tunneled subcutaneously, and anastomosed to the right axillary artery. Another 8-mm synthetic graft was utilized as a separate bypass from the axillary to the right common carotid artery.

Exposure and dissection of the mediastinum confirmed that the inflammatory process encased not only the innominate artery and innominate vein, but it also encircled the entirety of the aortic arch, mandating total arch replacement.

Cardiopulmonary bypass was established via cannulation of the ascending aorta, proximal to the perivascular phlegmon. The innominate vein and artery were divided, the latter at its bifurcation.

At a core temperature of 27°C, the circulation was arrested. The aortic arch was opened. The entire arch was involved in the inflammatory process, up to and beyond the origin of the left subclavian artery.

The left common carotid artery was divided and cannulated to facilitate unilateral selective antegrade cerebral perfusion (flow rate 10 mL/kg, titrated according to continuous noninvasive cerebral oximetry; our technique for this has been described previously). 3 The aortic arch and innominate artery were resected, as were the bases of the left common carotid and subclavian arteries. The left subclavian artery was ligated.

**Fig. 2:**
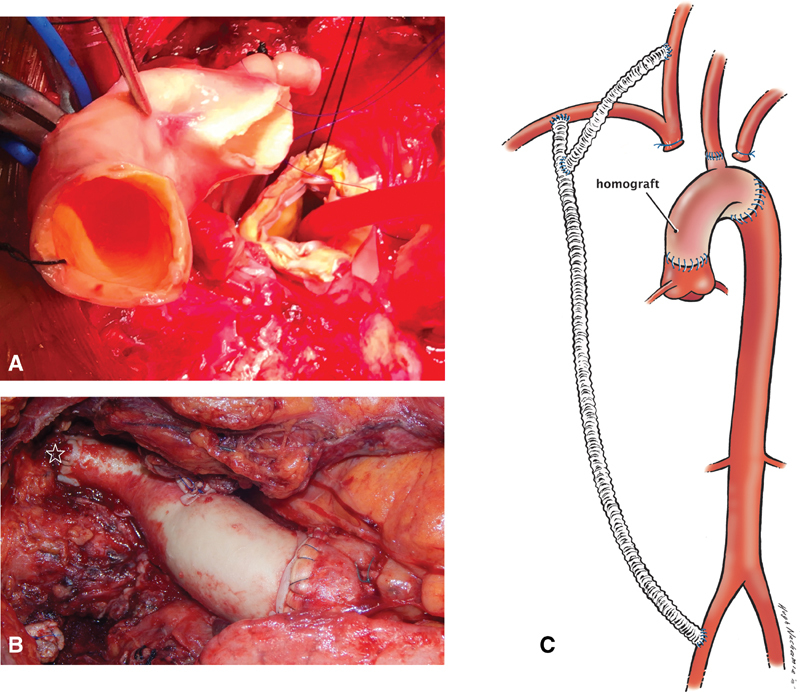
( **A** ) Homograft was used to fashion a new aortic arch. ( **B, C** ) The completed reconstruction. One of the homograft branches revascularized the left common carotid artery (star).

A 25-mm homograft (Cryolife, Inc., Kennesaw, GA) was anastomosed primarily to the proximal descending thoracic aorta ( Fig. 2A ). The homograft neoarch was cannulated and cardiopulmonary bypass was resumed. As the patient was rewarmed, one of the branches of the aortic homograft was used to revascularize the left common carotid artery within the chest. The proximal aortic anastomosis was fashioned between the homograft and midascending aorta to complete the reconstruction ( Fig. 2B, C ).

The patient was separated from cardiopulmonary bypass without complication. Total cardiopulmonary bypass time was 170 minutes. Aortic crossclamp time was 92 minutes. Circulatory arrest time was 41 minutes, with continuous unilateral antegrade cerebral perfusion maintained throughout.

The chest was left open. The patient returned to the operating room 24 hours later, whereupon a flap of greater omentum was harvested laparoscopically, brought through the diaphragm, and used to envelop the newly created aortic arch. The sternum was closed primarily.

The patient was discharged on a prolonged 8-week course of parenteral antibiotics. At 12 months of follow-up, the patient had no evidence of recurrent infection.

## Discussion

Although the role of extra-anatomic bypass in surgery for arch infection has been described previously, 4 this is the first report utilizing the femoral artery as inflow for a composite bypass to the right cerebral circulation. This sequential debranching procedure, completed prior to the aortic reconstruction, afforded us two major advantages.

First, by rerouting right cerebral blood flow outside the chest, we were able to avoid using prosthetic graft to reconstruct the innominate artery within the infected mediastinum. We were thus able to minimize the amount of foreign material implanted within the infected tissue space itself, reducing the patient's risk of recurrent infection, always a concern with in-line arch replacement in a contaminated surgical field.

Second, and perhaps more importantly, the composite right femoral–axillary–carotid bypass ensured that, at no stage, it was the brain devoid of antegrade perfusion, at least unilaterally. During hypothermic circulatory arrest, while there was no flow through the right carotid, the left cerebrum was perfused via direct cannulation of the left common carotid artery. Subsequently, after cardiopulmonary bypass had been reestablished, during the period of left carotid artery ischemia, the right carotid artery was continuously perfused via the previously constructed extrathoracic bypass network. These neuroprotective benefits are of paramount importance, given the inherent risk of neurological injury during arch surgery in a septic octogenarian patient.

Although the left subclavian artery could have been reattached to the homograft, rather than ligated, we nevertheless elected to transect this vessel to avoid the potential pitfalls of a technically challenging anastomosis deep within the chest in a grossly infected surgical field. Although not required by our patient, an extrathoracic, extraanatomic left carotid to subclavian bypass could be fashioned at any time where it deemed necessary.

Our patient's aortic arch infection was secondary to *S* . *aureus* . Although *Salmonella* aortitis is a well-recognized entity, particularly in the descending and abdominal aorta, 5 infections isolated to the aortic arch tend to have a more varied microbiologic etiology. In a review of nine patients with infected distal arch aneurysms, Okada and associates found *S* . *aureus* as the inciting pathogen in two cases, with a myriad of different organisms (all non- *Salmonella* species) identified in the other cases. 6 We were not able to definitively determine the source for the inoculum that caused our patient's infection, although we presume that she may have had a penetrating atherosclerotic ulcer (PAU) at the base of her innominate artery and proximal arch. Penetrating atherosclerotic ulcer has been proposed as the most common underlying cause of native aortic infection, 7 but whether this is true in all cases remains, as yet, open for discussion.

Primary infections of the aorta and great vessels require an individualized approach to management. Unusual extra-anatomic bypass grafts, 8 such as the configuration described by us, are useful to facilitate end-organ and cerebral perfusion, while concomitantly reducing the amount of prosthetic material implanted into an infected surgical field. Techniques such as this can be valuable to facilitate the reconstruction in these challenging cases.
